# Supplementary material for: Microbiota succession influences nematode physiology in a beetle microcosm ecosystem
Source: Nat Commun. 2024 Jun 15;15:5137. doi: 10.1038/s41467-024-49513-5 (PMC11180206; doi:10.1038/s41467-024-49513-5)
Supplement: Supplementary file 1 — Supplementary Information [file 41467_2024_49513_MOESM1_ESM.pdf]

Supplementary information

Microbiota succession influences nematode physiology in a beetle microcosm ecosystem

Lo et al.

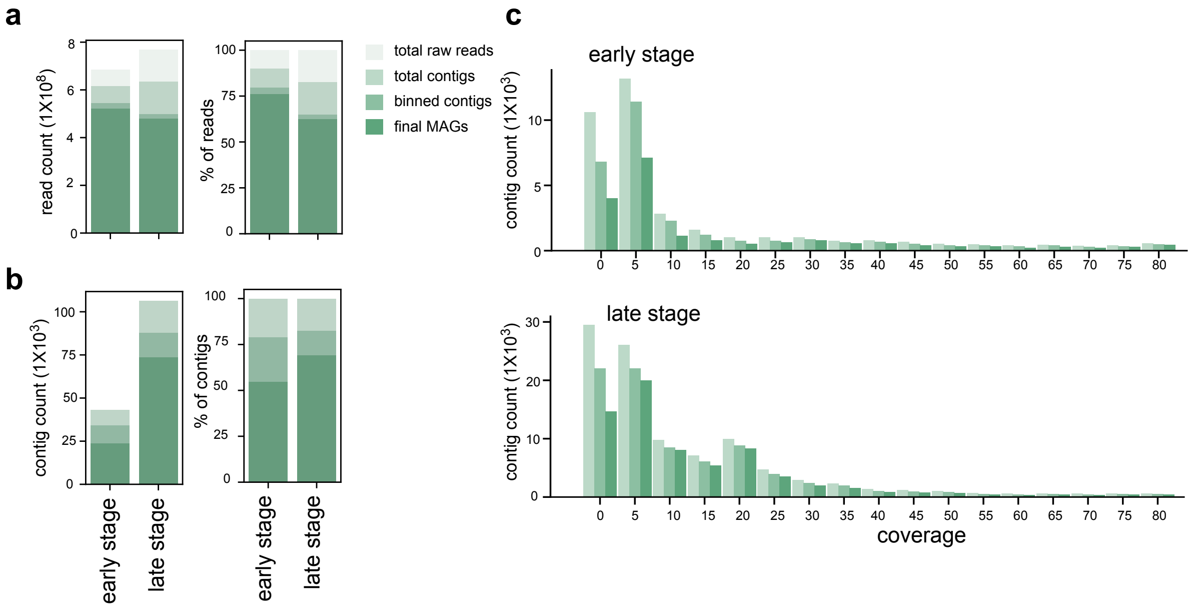

Supplementary Figure 1. Summary of raw reads utilisation and contig coverage in metagenome-assembled genomes (MAGs). (a) Overview of raw read counts and proportions at both early and late stages, detailing total raw reads, raw reads used in contig assembly, contigs subjected to binning, and contigs ultimately included in the final MAGs. (b) Total counts and relative proportions of total contigs, binned contigs, and contigs in the final MAGs. (c) Distribution of contig coverage, focusing on the 0-80 range, illustrating the prevalence of low-coverage contigs excluded from the final MAGs. c. Coverage distribution profile of contigs, illustrating the range from 0 - 90, to underscore the exclusion of low-coverage contigs from the final selection of MAGs.

Supplementary Figure 2

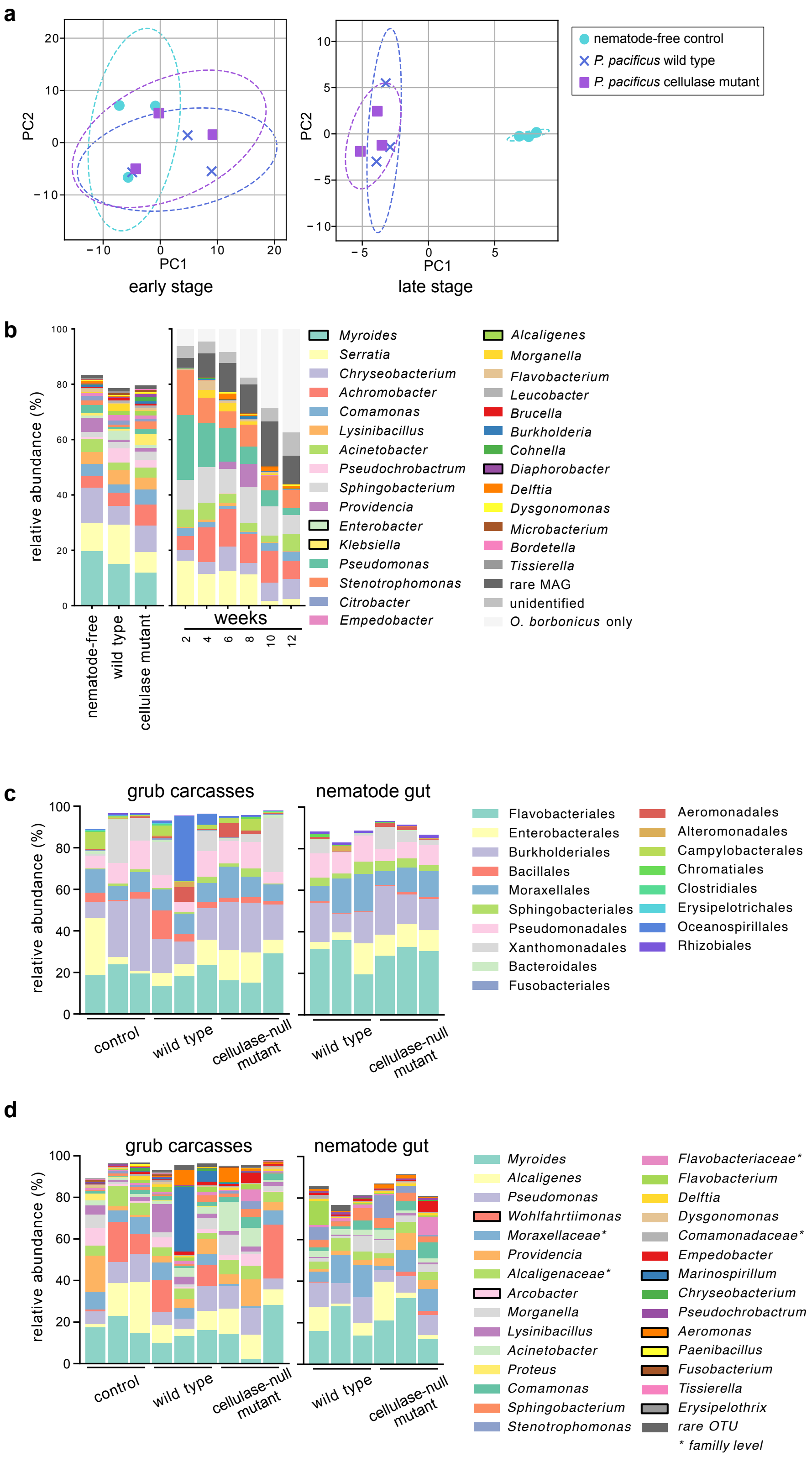

Supplementary Figure 2. Bacterial community profiles of decomposing rose chafer grubs, rhinoceros beetles, and white-spotted flower chafer. (a) Principal Component Analysis (PCA) of the bacterial community structure of each treatment at the early and late stage. Ellipses represent 95% confidence intervals for each treatment group. PERMANOVA indicates significant differences in community structure at the late stages. (b) Relative abundance of microbiota at the genus level of carcasses of rose chafer grubs (*Cetonia aurata*, left) from this study and that of rhinoceros beetle *Oryctes borbonicus* collected from La Réunion Island through the microbiota succession (right). Operational taxonomic units from *O. borbonicus* are arranged according to the ranking of the *C. aurata* grub sample. The percentages of abundances for the biological replicates were averaged to obtain the mean relative abundance. The squares with the black bolder line are bacterial genus specific to rose chafer. Relative abundance of order (c) and genus (d) level microbiota of white-spotted flower chafer carcasses and the gut microbiota of *P. pacificus* wild type and cellulase-null mutant derived from the carcasses. The squares with the black bolder line are bacterial genus specific to white-spotted flower and not found in rose chafer.

Supplementary Figure 3

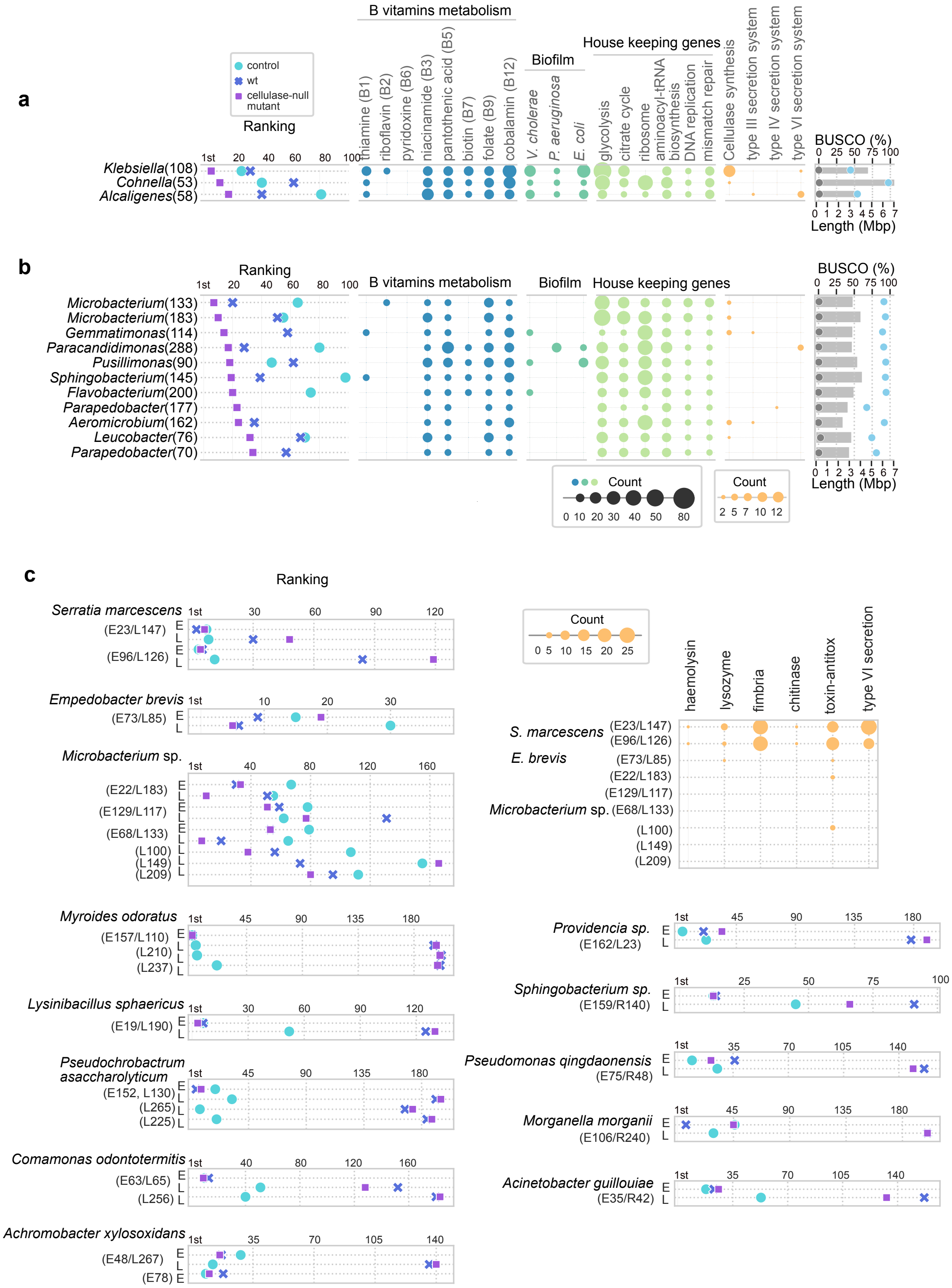

Supplementary Figure 3. Characters of metagenome-assembled genomes (MAGs) that are dominant in the presence of cellulase-null mutant *P. pacificus* in the early (a) and late (b) stage, and (c) the rankings and virulence genes of MAGs with identical taxonomic assignments observed across both time points. The abundance in ranking, gene numbers in metabolism pathways, genome size (grey bar) and the BUSCO completeness (blue dot) and contamination (grey dot) values are shown. The figures present the top 100 MAGs for both the early and late stages, followed by ranking from 1 to 220 for MAGs persisting across both stages. (c) The ranking of MAGs, with prefixes 'E' and 'L' to indicate their origin from the early or late stage, respectively. MAGs labelled with both 'E' and 'L' prefixes indicate they are identical assemblies from across early and late stages. The top-right corner detected genes potentially related to pathogenicity and competitive interactions.

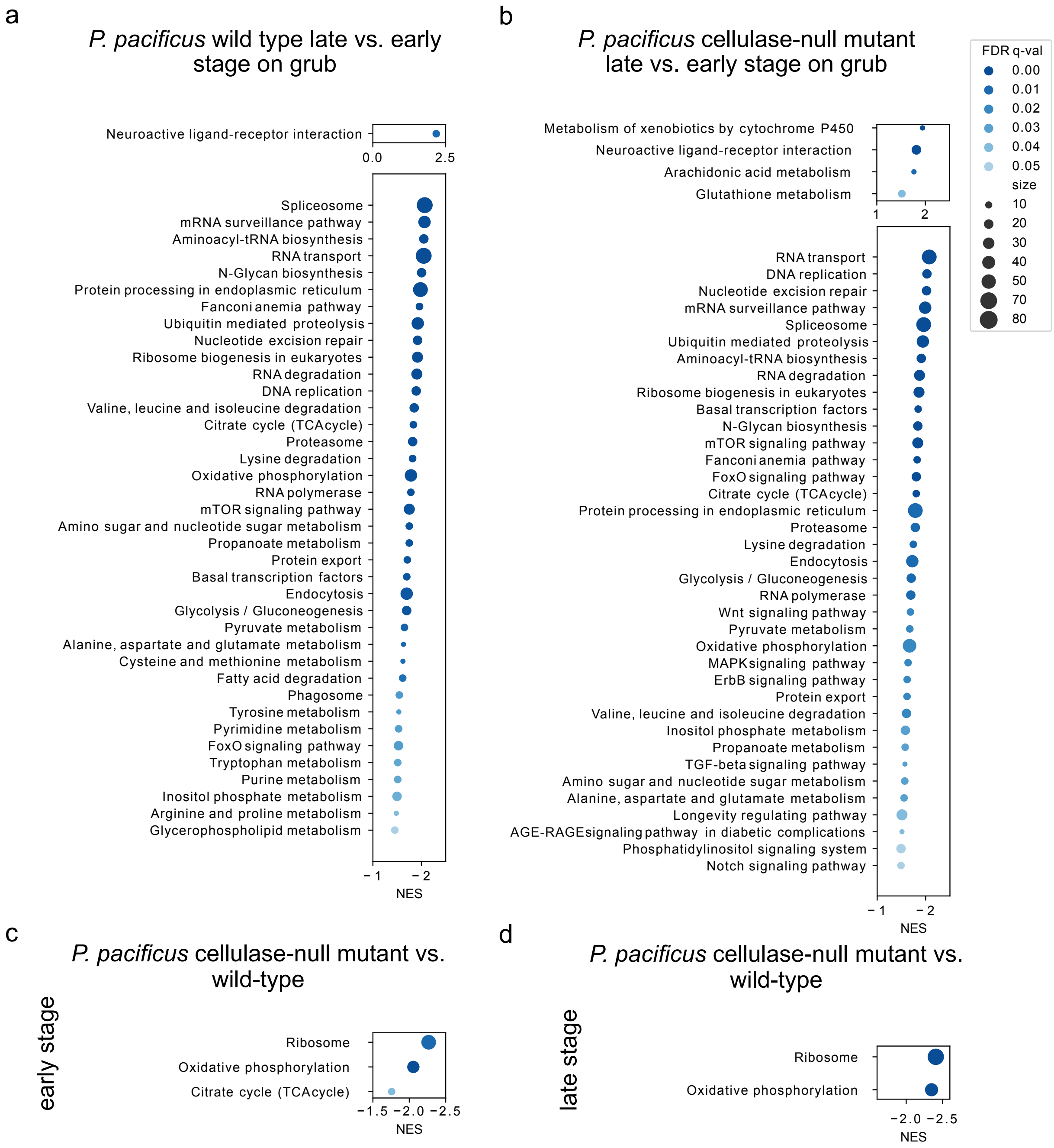

Supplementary Figure 4. Transcriptomic profiling of mixed stage *Pristionchus pacificus* wild type and cellulase-null mutant under different growing conditions. Cross comparisons between *P. pacificus* strains and rose chafer grubs (*Cetonia aurata*) decomposition stages using the Gene Set Enrichment Analysis (GSEA) via the KEGG gene set. Enriched pathways with a false discovery rate < 0.05 are presented and size indicates the number of genes in the pathway. (a) *P. pacificus* wild type at the early

stage vs. late stage. (b) cellulase-null mutant at the early stage vs. late stage. (c) cellulase-null mutant vs. wild type at the early stage and (d) at the late stage of decomposition.

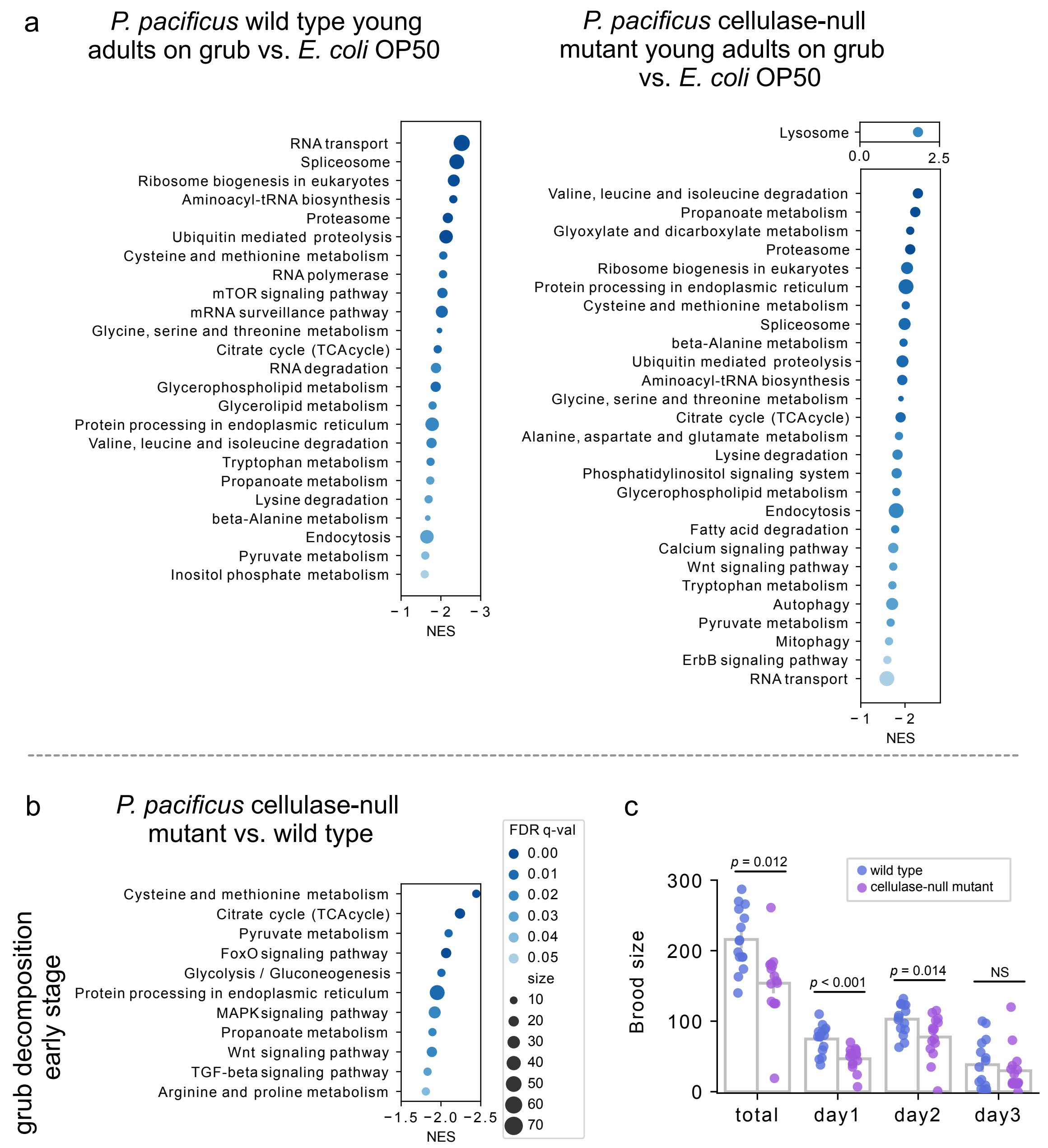

Supplementary Figure 5. Transcriptomic profiling of young adult *P. pacificus* wild type and cellulase-null mutant and brood size assessments. (a) Young adult nematodes reared on white-spotted flower chafer grub (*Protaetia brevitarsis*) carcasses were extracted at the early stage of decomposition (7 days after nematode inoculation). A Gene Set Enrichment Analysis (GSEA) was performed to detect the changed metabolic pathways via the KEGG gene set. Enriched pathways with a false discovery rate < 0.05 are presented and size indicates the number of genes in the pathway. (a)

Young adults of *P. pacificus* wild type reared on grub vs. *E. coli* OP50 (left) and young adults of cellulase-mutant on grub vs. *E. coli* OP50 (right). (b) A comparison between wild type and cellulase-null mutant at the early stage of decomposition. (c) The brood size of wild type or cellulase-null mutant individuals from the early stage of decomposition, n = 15. Source data are provided as a Source Data file.

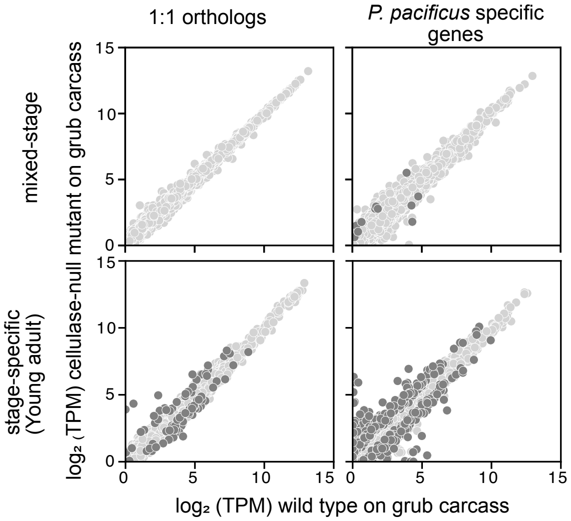

Supplementary Figure 6 Comparative gene expression and differentially expressed genes in *Pristionchus pacificus* wild type and cellulase-null mutant based on mixed-stage and stage-specific populations. The scatter plot illustrates the gene expression comparison between the wild type and cellulase-null mutant strains collected from grub carcasses at the early stage. For the stage-specific population analysis, young adult nematodes were used. Dark grey dots indicate the differentially expressed gene detected by DESeq2.
