# Supplementary material for: Microbiota succession influences nematode physiology in a beetle microcosm ecosystem
Source: Nat Commun. 2024 Jun 15;15:5137. doi: 10.1038/s41467-024-49513-5 (PMC11180206; doi:10.1038/s41467-024-49513-5)
Supplement: Supplementary file 3 — Description of Additional Supplementary Files [file 41467_2024_49513_MOESM3_ESM.pdf]

Description of Additional Supplementary Files

Supplementary Data 1. Characterization of metagenome-assembled genomes.

Supplementary Data 2. Gene Set Enrichment Analysis (GSEA) results summary. This supplementary table is organised into multiple sheets, each named according to the specific treatment comparison being presented. The naming convention for each sheet comprises three parts: the gene category used in GSEA, the *Pristionchus pacificus* strain, and the environmental context. For instance, 'KEGG_ps312_op50ES' refers to a comparison involving the KEGG gene category, the wild type *P. pacificus* strain (ps312), and the early stage (ES) of the *E. coli* OP50 environment. Similarly, other sheets follow this pattern, encompassing comparisons with the cellulase-null mutant (tu1539) across different environmental conditions like *E. coli* OP50, early stage (ES), and late stage (LS) of decomposed grub. Statistical analysis was performed using a permutation test, with results adjusted for multiple comparisons using the False Discovery Rate (FDR).

Supplementary Data 3. Transcripts per million (TPM) in transcriptome analysis of Pristionchus pacificus wild type and cellulase-null mutant.
