# Supplementary material for: Microbiota succession influences nematode physiology in a beetle microcosm ecosystem
Source: Nat Commun. 2024 Jun 15;15:5137. doi: 10.1038/s41467-024-49513-5 (PMC11180206; doi:10.1038/s41467-024-49513-5)
Supplement: Supplementary file 7 — Reporting Summary [file 41467_2024_49513_MOESM7_ESM.pdf]

nature portfolio

Corresponding author(s): Ralf J. Sommer and Ziduan Han

Last updated by author(s): May 15, 2024

Reporting Summary

Nature Portfolio wishes to improve the reproducibility of the work that we publish. This form provides structure for consistency and transparency in reporting. For further information on Nature Portfolio policies, see our Editorial Policies and the Editorial Policy Checklist.

Statistics

For all statistical analyses, confirm that the following items are present in the figure legend, table legend, main text, or Methods section.

n/a	Confirmed	
☐	☒	The exact sample size (n) for each experimental group/condition, given as a discrete number and unit of measurement
☐	☒	A statement on whether measurements were taken from distinct samples or whether the same sample was measured repeatedly
☐	☒	The statistical test(s) used AND whether they are one- or two-sided<br>*Only common tests should be described solely by name; describe more complex techniques in the Methods section.*
☒	☐	A description of all covariates tested
☒	☐	A description of any assumptions or corrections, such as tests of normality and adjustment for multiple comparisons
☐	☒	A full description of the statistical parameters including central tendency (e.g. means) or other basic estimates (e.g. regression coefficient) AND variation (e.g. standard deviation) or associated estimates of uncertainty (e.g. confidence intervals)
☐	☒	For null hypothesis testing, the test statistic (e.g. F, t, r) with confidence intervals, effect sizes, degrees of freedom and P value noted<br>*Give P values as exact values whenever suitable.*
☒	☐	For Bayesian analysis, information on the choice of priors and Markov chain Monte Carlo settings
☒	☐	For hierarchical and complex designs, identification of the appropriate level for tests and full reporting of outcomes
☒	☐	Estimates of effect sizes (e.g. Cohen's d, Pearson's r), indicating how they were calculated

Our web collection on statistics for biologists contains articles on many of the points above.

Software and code

Policy information about availability of computer code

Data collection	Our data were collected through researcher observations and NGS sequencing analysis, without the use of software for initial data acquisition.
Data analysis	Data analysis were carried out using software HISAT2 v2.1.0, StringTie2 v2.1.7, featureCounts v1.6.3, OrthoFinder v2.5.5, DESeq2 v1.42.0 for RNA-seq experiments. muthor v1.44.0, metasPAdes v3.15.3, MetaBAT2 v2.12.1, BUSC0 v5.3.2, eggN0G-mapper v2 and diamond v2.0.13.151 for metagenomics studies

For manuscripts utilizing custom algorithms or software that are central to the research but not yet described in published literature, software must be made available to editors and reviewers. We strongly encourage code deposition in a community repository (e.g. GitHub). See the Nature Portfolio guidelines for submitting code & software for further information.

Data

Policy information about availability of data

All manuscripts must include a data availability statement. This statement should provide the following information, where applicable:

- Accession codes, unique identifiers, or web links for publicly available datasets
- A description of any restrictions on data availability
- For clinical datasets or third party data, please ensure that the statement adheres to our policy

Sequencing data have been deposited at NCBI Sequence Read Archive, and accession numbers are PRJNA938361 and PRJNA938905. Scripts and source data used to generate figures in this study are available in Zenodo (https://zenodo.org/records/11196560).

Research involving human participants, their data, or biological material

Policy information about studies with human participants or human data. See also policy information about sex, gender (identity/presentation), and sexual orientation and race, ethnicity and racism.

Reporting on sex and gender	This study did not involve research on human participants, their data, or biological material. Therefore, this section is not applicable to our study.
Reporting on race, ethnicity, or other socially relevant groupings	This study did not involve research on human participants, their data, or biological material. Therefore, this section is not applicable to our study.
Population characteristics	This study did not involve research on human participants, their data, or biological material. Therefore, this section is not applicable to our study.
Recruitment	This study did not involve research on human participants, their data, or biological material. Therefore, this section is not applicable to our study.
Ethics oversight	This study did not involve research on human participants, their data, or biological material. Therefore, this section is not applicable to our study.

Note that full information on the approval of the study protocol must also be provided in the manuscript.

Field-specific reporting

Please select the one below that is the best fit for your research. If you are not sure, read the appropriate sections before making your selection.

☒ Life sciences ☐ Behavioural & social sciences ☐ Ecological, evolutionary & environmental sciences

For a reference copy of the document with all sections, see nature.com/documents/nr-reporting-summary-flat.pdf

Life sciences study design

All studies must disclose on these points even when the disclosure is negative.

Sample size	we utilized tens of thousands of individuals from laboratory type strains of nematodes Pristionchus pacificus PS312 and their respective cellulase mutant. These individuals were grouped into appropriate batches to represent different experimental conditions or groups.
Data exclusions	We did not exclude any data or samples. All collected data were subjected to analysis and are presented in the results
Replication	Each treatment requiring NGS sequencing and Oil-Red-O staining was conducted with three biological replicates. Experiments involving dauer formation were replicated six times.
Randomization	This study did not involve randomization
Blinding	This study did not involve blinding

Reporting for specific materials, systems and methods

We require information from authors about some types of materials, experimental systems and methods used in many studies. Here, indicate whether each material, system or method listed is relevant to your study. If you are not sure if a list item applies to your research, read the appropriate section before selecting a response.

Materials & experimental systems

n/a	Involved in the study	
☒	☐	Antibodies
☒	☐	Eukaryotic cell lines
☒	☐	Palaeontology and archaeology
☐	☒	Animals and other organisms
☒	☐	Clinical data
☒	☐	Dual use research of concern
☒	☐	Plants

Methods

n/a	Involved in the study	
☒	☐	ChIP-seq
☒	☐	Flow cytometry
☒	☐	MRI-based neuroimaging

Animals and other research organisms

Policy information about studies involving animals; ARRIVE guidelines recommended for reporting animal research, and Sex and Gender in Research

Laboratory animals	Pristionchus pacificus
Wild animals	This study did not involve wild animals
Reporting on sex	This study utilized the P. pacificus nematode, a predominantly hermaphroditic system with about 1% of the population spontaneously developing as males.
Field-collected samples	This study was conducted using laboratory model nematodes and commercially available beetle grubs; no sample was collected from the wild.
Ethics oversight	This study involving nematodes and beetle grubs do not require ethics oversight

Note that full information on the approval of the study protocol must also be provided in the manuscript.
